# Functional-Structural Coupling: Brain Reorganization in Presbycusis Is Related to Cognitive Impairment

**DOI:** 10.1523/ENEURO.0294-25.2026

**Published:** 2026-03-03

**Authors:** Xiaojie Li, Weilong Fu, Yao Wang, Yuting Gao, Jinhai Wang, Jing Yang, Longji Xu, Fei Gao, Xiao Li, Ning Li

**Affiliations:** ^1^School of Life Sciences, Tiangong University, Tianjin 300380, China; ^2^Tianjin Key Laboratory of Optoelectronic Detection Technology and System, Tianjin 300387, China; ^3^Department of Radiology, Shandong Provincial Hospital Affiliated to Shandong First Medical University, Jinan 271016, China

**Keywords:** brain reorganization, cognitive impairment, fMRI, presbycusis, sMRI

## Abstract

Presbycusis, a prevalent neurodegenerative disorder, is characterized by declining speech recognition and has been associated with cognitive impairments across multiple domains. However, the underlying neurobiological mechanisms between presbycusis and cognitive impairments remain unclear. We assessed pure-tone audiometry thresholds (PTA), speech recognition thresholds (SRT), and cognitive abilities in individuals with presbycusis (24 males and 31 females) and healthy controls (23 males and 32 females). Using magnetic resonance imaging, we calculated the amplitude of low-frequency fluctuations (ALFF) to characterize function and gray matter volume (GMV) to characterize structure. Based on ALFF and GMV, we calculated functional-structural ratio (FSR) to measure the functional-structural coupling. Significant correlations between GMV atrophy and ALFF changed in the putamen, fusiform gyrus, precuneus, and medial superior frontal gyrus in presbycusis group, and these changes were significantly associated with the increase in PTA and SRT. The FSR reduction in the FFG, precuneus, and medial superior frontal gyrus were also significantly associated with the increase in PTA and SRT. Moreover, it was also significantly correlated with lower scores on the Montreal Cognitive Assessment (MoCA) and the Auditory Verbal Learning Test (AVLT), as well as the prolonged time in the Trail Making Test (TMT-A). Presbycusis involves coupled structural atrophy and functional decline in auditory and higher-order cognitive regions. Crucially, reduced FSR correlates with both worsening hearing thresholds and cognitive impairment. This highlights FSR as a key neurobiological link between hearing loss and cognitive decline. This research provides a novel basis for early screening and dynamic monitoring of presbycusis-related cognitive impairment.

## Visual Abstract

**Figure:**
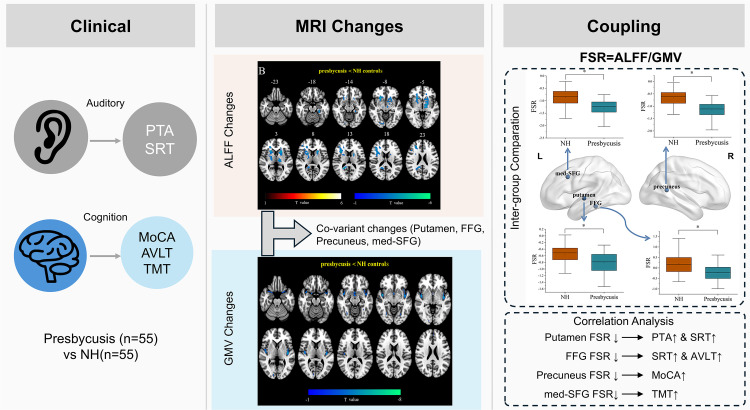

## Significance Statement

This study reveals that age-related hearing loss (presbycusis) involves coupled structural atrophy and functional decline in key brain regions like the fusiform gyrus and putamen. We introduce the functional-structural ratio (FSR) as a novel biomarker showing that reduced brain functional-structural coupling correlates with both worsening hearing thresholds and cognitive impairment. This provides the first direct neurobiological evidence linking hearing loss to cognitive decline via shared neural reorganization. FSR offers a potential tool for early screening and monitoring of dementia risk in presbycusis, highlighting that preserving hearing health may protect brain integrity. These findings advance our understanding of how sensory decline drives neurodegeneration.

## Introduction

Presbycusis, a common condition in older adults, is characterized by peripheral damage and central brain damage (Uchida et al., 2011). In most instances, presbycusis presents as bilateral hearing loss (HL) accompanied by a decline in speech recognition ability (Lin et al., 2011). According to a 2021 report by the World Health Organization, >65% of individuals aged 60 and above globally experience varying degrees of hearing impairment (World Health Organization, 2021). It is worth noting that HL has been identified as the primary modifiable risk factor for dementia (Livingston et al., 2017). Numerous studies have increasingly linked presbycusis with cognitive impairments in multiple domains (Jafari et al., 2019; Wang et al., 2022). Notably, a regression analysis has demonstrated that hearing thresholds can serve as predictive indicators for executive function deficits in individuals with presbycusis (Viñas-Guasch and Wu, 2017). Another study suggested that age-related HL may be compensated for by recruiting working memory (Rosemann and Thiel, 2020). A recent study combined pure-tone average (PTA) and distortion product otoacoustic emissions to evaluate the relationship between auditory and cognitive impairments in presbycusis (Medel et al., 2024). However, the underlying neural mechanisms linking presbycusis and cognitive impairments remain under investigation.

In recent times, there has been a widespread use of magnetic resonance imaging (MRI) technology to examine the pathogenesis of diverse neurological and psychiatric diseases. Gray matter volume (GMV), a metric derived from structural MRI (sMRI), is utilized to quantitatively measure the amount of gray matter in the brain. It is a method of characterizing the number of neurons and qualitatively assessing GMV based on T1-weighted image intensity. A previous study revealed that presbycusis is associated with reduced GMV in the temporal lobe, which correlates with functional impairment (Belkhiria et al., 2020). In another study, this functional impairment was further elucidated as cognitive deterioration, specifically establishing a correlation between reduced GMV in the prefrontal cortex and diminished attention in presbycusis (Ren et al., 2018). A resting-state functional MRI (fMRI) metric called the amplitude of low-frequency fluctuations (ALFF) is used to characterize the intensity of spontaneous neural activity within specific brain regions. Presbycusis hearing loss is not only the functional decline of peripheral auditory organs but also involves the reorganization of the central auditory pathway and cross-modal brain networks. ALFF provides a key tool for revealing its neural mechanism. A study has reported that presbycusis exhibits heightened ALFF in the Heschl's gyrus (HG), which demonstrates a positive correlation with Auditory Verbal Learning Test (AVLT) scores (Ren et al., 2021). In summary, previous studies have consistently demonstrated reorganization at both the functional and structural levels in presbycusis, respectively, which is closely associated with cognitive impairments.

However, recent studies have revealed an interdependent relationship between functional and structural reorganizations observed in neurodegenerative diseases. For instance, a study investigating cognitive impairment in Parkinson's disease reported a correlation between temporal lobe atrophy and enhanced ALFF (Zheng et al., 2022). A recent study utilizing deep learning techniques demonstrated that the integration of sMRI and fMRI data yields superior predictive performance for Alzheimer's disease (Zhou et al., 2018). However, these algorithmic studies primarily focus on disease prediction, while the underlying mechanisms of reorganization within specific brain regions remain elusive. Moreover, there is currently a lack of comprehensive investigations exploring the pathological mechanisms of reorganization by integrating both functional and structural aspects of presbycusis.

Recent advances in multimodal neuroimaging have highlighted the importance of investigating the functional-structural coupling to better understand neural reorganization associated with aging and sensory processing. Traditional coupling analyses such as correlations or regressions between GMV and resting-state functional measures capture global associations but may fail to reveal localized imbalances between functional activity and the underlying structural substrate. To address this limitation, several recent studies have introduced ratio-based coupling indices that quantify functional activity relative to tissue volume, such as the amplitude of ALFF divided by GMV or voxel-based morphometry (VBM) estimates (Zhang et al., 2023; Zhao et al., 2023; Wang et al., 2025). These metrics have been interpreted as reflecting the “neural activity demand per unit of gray matter volume” (Zhang et al., 2023), providing a biologically meaningful measure of local functional-structural coupling. Abnormally high or low values of such ratios may indicate functional-structural decoupling, where neural activity is either disproportionate to or constrained by regional structural integrity. Following this rationale, we defined a functional-structural ratio (FSR)—calculated as ALFF divided by GMV—to characterize ROI-wise functional-structural coupling alterations in presbycusis. This approach allows spatially specific assessment of functional-structural relationships and may reveal compensatory or maladaptive reorganizations not captured by traditional correlation-based methods.

To this end, this study aimed to investigate the neural mechanisms underlying cognitive impairments in presbycusis by combining structural MRI (sMRI) and functional MRI (fMRI). The Montreal Cognitive Assessment (MoCA), Auditory Verbal Learning Test (AVLT), and Trail Making Test (TMT) were administered to assess cognitive function in patients with presbycusis. To quantify the relationship between regional brain activation and gray matter volume, we applied FSR as a measure of functional-structural coupling. Using this metric, we further examined whether alterations in coupling occur in presbycusis and how these changes relate to cognitive performance. In addition, moderation analysis was conducted to explore the interrelationships among functional-structural coupling, cognitive function, and hearing damage severity.

## Materials and Methods

### Participants

A total of 110 participants (63 females, 47 males) aged from 50 to 74 years (mean ± standard deviation = 64.27 ± 4.15 years) were enrolled in this study, including 55 presbycusis patients and 55 normal hearing (NH) controls. Each participant was right-handed and a speaker of Mandarin Chinese. Participants with a PTA > 25 decibels hearing level (dB HL) at four frequencies (0.5, 1, 2, and 4 kHz) were included in the presbycusis group. The presbycusis group consists of 35 mild HL patients, 19 moderate HL patients, and 1 severe HL patient. The exclusion criteria were as follows: (1) cause of HL other than presbycusis; (2) history of otologic surgery and use of hearing aid; (3) neurological disease; and (4) contraindications for MRI. All participants of this study provided written informed consent.

### Auditory and cognitive function test

In a sound-attenuating booth, participants completed an auditory evaluation while remaining vigilant throughout the entire time. A Madsen Electronics Midimate 622 audiometer was used to test the pure-tone thresholds at frequencies ranging from 0.125 to 8 kHz in both ears. The speech reception threshold (SRT; Schlauch et al., 1996) was assessed by the HOPE program (Schlauch et al., 1996) to evaluate speech recognition capabilities. The Montreal Cognitive Assessment (MoCA; Nasreddine et al., 2005) was employed to assess general cognitive function. The AVLT was utilized to evaluate auditory verbal memory performance. Additionally, the Trail Making Test (TMT; Sánchez-Cubillo et al., 2009) is divided into A and B. TMT-A was used to assess executive function, whereas TMT-B was used to assess control capacity.

### MRI data acquisition

All MRI data acquisitions were performed on a 3.0 T scanner (Philips, Achieva) using an eight-channel phased-array head coil. The sMRI data were obtained using T1-weighted 3D TFE sequence (TR/TE, 8.1/3.7 ms; thickness, 1 mm; voxel size, 1 × 1 × 1 mm^3^; field of view, 24 × 24 cm^2^; turning angle, 8°; 160 slices). The fMRI data were obtained using an echoplanar sequence (TR/TE, 2,000/35 ms; resolution, 3.75 × 3.75 mm^2^; slice thickness, 4 mm; field of view, 24 × 24 cm^2^; 35 slices; 240 dynamic), which lasted for 8 min. During the scan, the participants were instructed to lie quietly and keep still, with eyes closed but not asleep or think about anything special. Foam pads and earplugs were used to minimize head movement and reduce noise.

### MRI data preprocessing

Structural image preprocessing was conducted utilizing Statistical Parametric Mapping software (SPM12; Friston, 2007) within the MATLAB environment (MathWorks). This process involved several preprocessing steps. Initially, each image was normalized to the Montreal Neurological Institute (MNI) space and subsequently segmented into white matter, gray matter, and cerebrospinal fluid (Ashburner, 2007). Gray matter volume (GMV) was extracted using a customized DARTEL template, with gray matter data resampled to 1.5 × 1.5 × 1.5 mm^3^ voxels. To improve the signal-to-noise ratio, the images underwent smoothing with a 4 mm Gaussian kernel. A threshold of 0.2 was implemented to mitigate edge effects between white and gray matter (Zhong et al., 2021). Ultimately, voxel-wise GMV data for the entire brain were acquired.

The fMRI data were preprocessed using the Data Processing Assistant for RS-fMRI (DPARSF; Chao-Gan and Yu-Feng, 2010; Yan et al., 2016), following the steps: First, the initial 10 volumes were discarded to ensure a stable magnetic field. The remaining data were corrected for slice-timing differences and head motion (excluding subjects with head motion >3 mm or head rotation >3°). Following this, nuisance signals from white matter and cerebrospinal fluid were regressed out to reduce respiratory and cardiac effects. The denoised functional data were then normalized to MNI space using DARTEL algorithm (Ashburner, 2007), resampled to a resolution of 3 × 3 × 3 mm^3^, and smoothed with a Gaussian kernel of 4 mm FWHM. Additionally, linear trends were removed from the data. Finally, a bandpass filter (0.01–0.1 Hz) was applied to extract low-frequency fluctuations (Zhong et al., 2024).

### Demographic and MRI analysis

Statistical analysis was performed using SPSS to analyze demographic information, auditory, and cognitive scale scores. The gender ratio was assessed using a chi-square test, while other comparisons were conducted using two-tailed two-sample *t* tests. Differences between groups were considered significant when *p* < 0.05.

Intergroup differences in ALFF and GMV between the presbycusis and NH groups were analyzed using two-sided two-sample *t* tests, with gender, age, and education level as covariates in all models. For GMV analyses, total intracranial volume (TIV) was additionally included as a covariate to account for variations in brain size. A significance level of *p* < 0.05 was applied after adjusting for multiple comparisons using the false discovery rate (FDR) method.

### Functional-structural coupling analysis

Initially, regions of interest (ROIs) were defined as the brain areas exhibiting covariant changes in both ALFF and GMV analyses. Within these ROIs, a partial correlation analysis was conducted to examine the direct relationship between ALFF and GMV, while controlling for age, gender, and education level (FDR, *p* < 0.05). To quantify functional-structural coupling, FSR was calculated for each participant by dividing the mean ALFF signal by the mean GMV (Cui et al., 2022). Subsequently, we compared the differentiation of FSR between the presbycusis and NH groups by two-tailed two-sample *t* tests (Bonferroni, *p* < 0.01). Finally, partial correlation analysis was conducted to explore the association between FSR and clinical variables in presbycusis group, which controlled for gender, age, and education level (*p* < 0.05).

## Results

### Demographic data

Table 1 illustrates all participants’ demographic and clinical characteristics. Age, gender, and educational attainment are not significantly different between the presbycusis and NH groups (*p* > 0.05). The PTA and SRT of the presbycusis group were significantly greater than those of the NH group (*p* < 0.001). All results on the cognitive evaluation scales showed significant group differences (*p* < 0.001). According to clinical standards, the presbycusis group consists of 19 presbycusis patients with normal cognition and 36 presbycusis patients with dementia (26 of whom have mild cognitive impairment).

**Table 1.** Participants’ demographic and clinical data

Characteristics	Presbycusis group (*n* = 55)	Normal hearing group (*n* = 55)	*p* value
Gender (male/female)	24/31	23/32	0.709
Age (years)	64.77 ± 4.61	63.78 ± 3.59	0.118
Education (years)	11.28 ± 2.74	11.39 ± 3.49	0.815
Disease duration (years)	3.10 ± 1.94	-	-
PTA (dB/HL)	38.55 ± 11.14	11.76 ± 3.99	<0.001*
SRT (dB/HL)	40.00 ± 15.43	13.22 ± 4.67	<0.001*
MoCA	22.38 ± 5.34	26.54 ± 2.97	<0.001*
AVLT	44.23 ± 12.12	51.33 ± 11.30	<0.001*
TMT-A(s)	85.10 ± 38.35	61.03 ± 24.79	<0.001*
TMT-B(s)	232.35 ± 85.70	157.31 ± 50.00	<0.001*

The data are presented as mean ± standard deviations. The bold and asterisked (*) values are statistically significant. PTA, pure tone average; SRT, speech reception threshold; MoCA, Montreal Cognitive Assessment; AVLT, Auditory Verbal Learning Test; TMT, Trail Making Test.

### Group differences in GMV and ALFF

**Figure 1.**
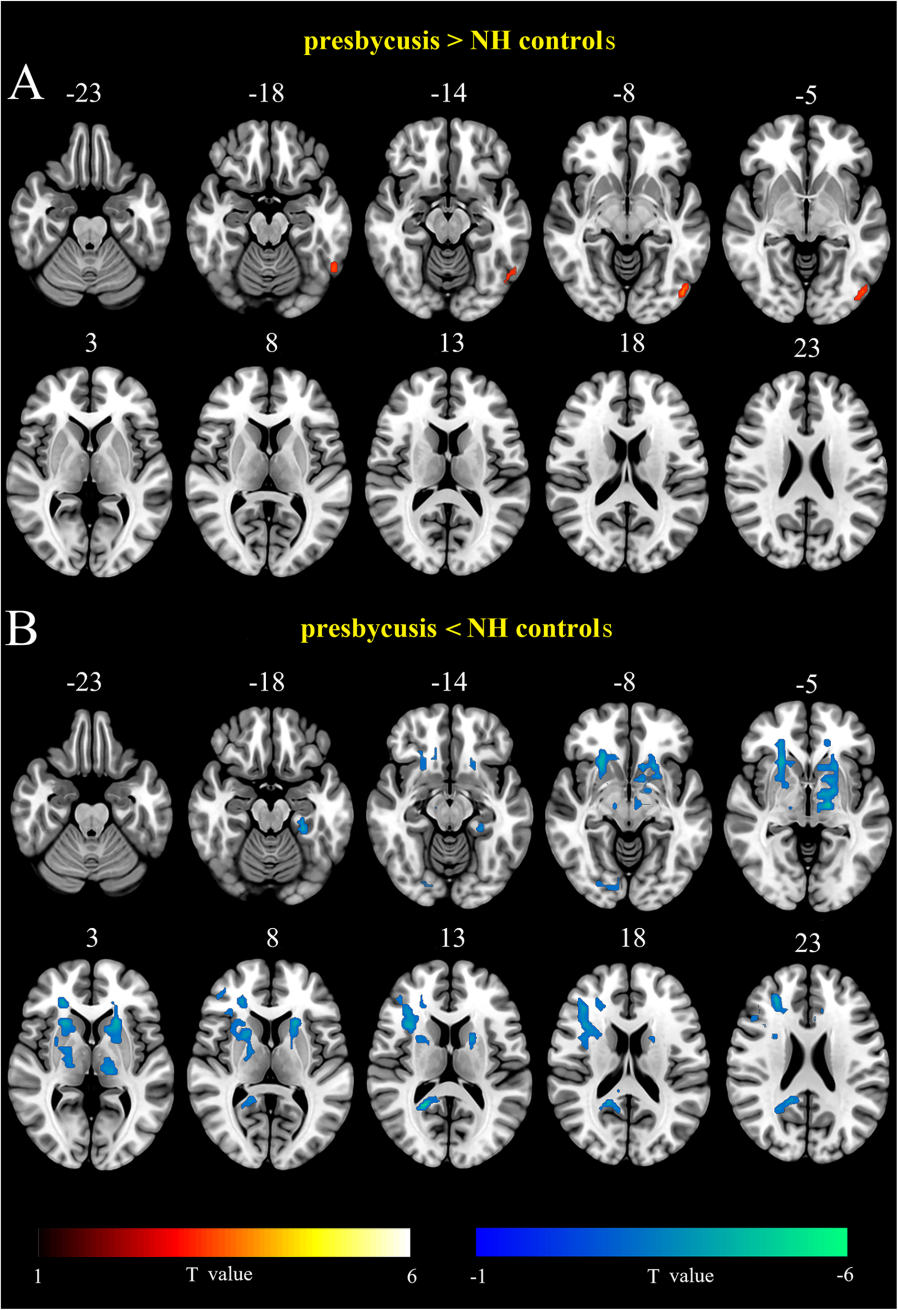
Visualization of intergroup differences in ALFF. Results were obtained by a two-tailed two-sample *t* test. FDR corrected *p* < 0.05, cluster size >20 voxels. L, left; R, right.

Figure 1 and Table 2 illustrate the group differences in ALFF for presbycusis and NH control. The bilateral superior frontal gyrus (SFG), middle frontal gyrus, inferior frontal gyrus, and supplementary motor area (SMA) all showed reduced ALFF in presbycusis when compared with the NH controls. In addition, presbycusis exhibited reduced ALFF in the right lingual gyrus, precuneus, left fusiform gyrus (FFG), middle cingulate cortex (MCC), medial superior frontal gyrus (med-SFG), putamen, and middle occipital gyrus. However, presbycusis exhibited increased ALFF in the right inferior temporal gyrus (ITG), the left calcarine, and the inferior occipital gyrus (IOG).

**Table 2.** The difference brain region of ALFF between presbycusis and normal hearing groups

Brain region	AAL Atlas	MNI coordinate			*T* value	Cluster size
		*x*	*y*	*z*		
Temporal_Inf_R	90	33	3	−48	4.7409	72
Calcarine_L	43	0	99	3	4.0657	65
Occipital_Inf_L	53	−48	−78	−6	4.1752	40
Fusiform_L	55	−27	−36	−18	−4.4788	22
Lingual _R	48	12	−87	−9	−3.7288	21
Frontal_Mid_2_R; Frontal_Sup_2_R; Frontal_Inf_Tri_R	4;8;14	27	36	27	−5.2274	643
Putamen_L	73	−18	−15	−3	−5.0439	320
Precuneus_R	68	21	−51	12	−5.496	142
Frontal_Inf_Oper_L	11	−39	12	12	−3.9399	23
Frontal_Sup_2_L; Frontal_Mid_2_L; Frontal_Sup_Medial_L	3;7;23	−12	15	36	−4.7907	391
Frontal_Inf_Oper_R	10	27	6	27	−3.9076	30
Occipital_Mid_L	51	−21	−60	33	−4.6049	26
Cingulate_Mid_L	33	−6	−27	36	−3.8781	29
Supp_Motor_Area_L	19;20	−9	9	60	−5.9602	66
Supp_Motor_Area_R						

FDR corrected *p* < 0.05, cluster size >20 voxels. ALFF, amplitude of low-frequency fluctuation; MNI, Montreal Neurological Institute; L, left; R, right.

**Figure 2.**
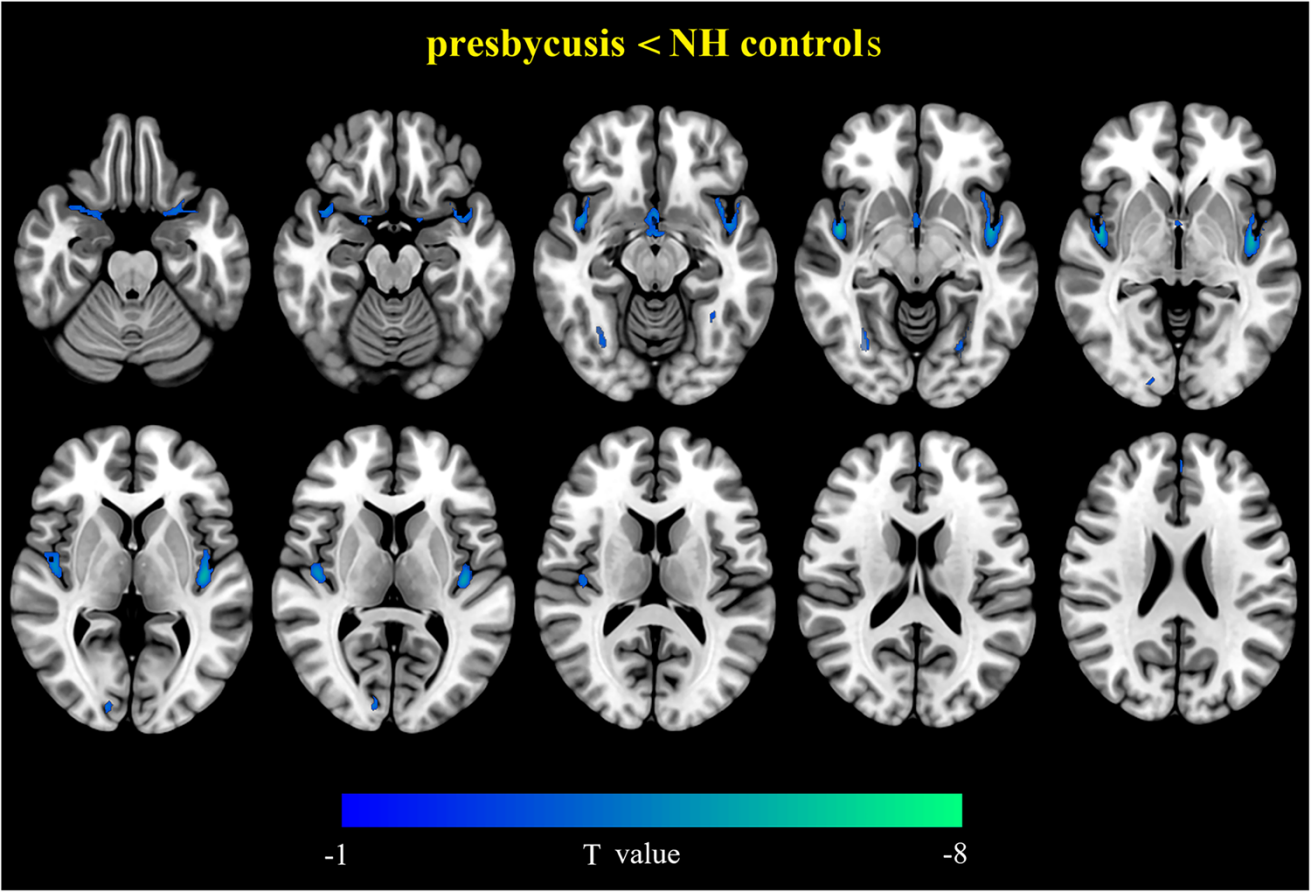
Visualization of intergroup differences in GMV. Results were obtained by a two-tailed two-sample *t* test. FDR corrected *p* < 0.05, cluster size >50 voxels. L, left; R, right.

Figure 2 and Table 3 illustrate the group differences in GMV for presbycusis and NH control. The bilateral superior temporal gyrus, superior temporal pole, MCC, insula, HG, FFG, and SMA all showed reduced GMV in the presbycusis when compared with the NH controls. In addition, presbycusis exhibited reduced GMV in the right middle temporal gyrus, hippocampus, calcarine, precuneus, left putamen, med-SFG, and inferior parietal lobule.

**Table 3.** The difference brain region of GMV between presbycusis and normal hearing groups

Brain region	AAL atlas	MNI coordinate			*T* value	Cluster size
		*x*	*y*	*z*		
Temporal_Mid_R	86	45	3	−34.5	−5.0268	51
Insula_L; Temporal_Sup_L; Temporal_Pole_Sup_L; Heschl_L	29;79;81;83	−43.5	−4.5	−4.5	−6.5406	1,102
Insula_R; Temporal_Sup_R; Temporal_Pole_Sup_R; Heschl_R	30;80;82;84	45	−1.5	−6	−6.7431	1,116
Fusiform_R	56	31.5	−64.5	−9	−6.3815	99
Fusiform_L	55	−25.5	−70.5	−7.5	−4.2432	57
Hippocampus_R	38	30	−25.5	−7.5	−4.2745	56
Calcarine_R	44	12	−90	7.5	−4.5464	83
Putamen_L	73	−31.5	−15	7.5	−5.0758	183
Frontal_Sup_Medial_L	23	−1.5	51	27	−4.1942	50
Cingulate_Mid_R; Cingulate_Mid_L	33;34	0	24	40.5	−4.4575	118
Parietal_Inf_L	61	−36	−54	51	−4.7037	62
Precuneus_R; Cingulate_Mid_R; Cingulate_Mid_L	33;34;68	4.5	−40.5	54	−4.2288	70
Supp_Motor_Area_R; Supp_Motor_Area_L	19;20	1.5	−1.5	54	−4.5935	98

FDR corrected *p* < 0.05, cluster size >50 voxels. GMV, gray matter volume; Montreal Neurological Institute; L, left; R, right.

### Correlation between ALFF and GMV in covariant regions

The correlation between ALFF and GMV in the same brain region of the presbycusis group is illustrated in Table 4. A significant negative correlation was observed between ALFF and GMV in the left SFG (*r* = −0.320, *p* = 0.006), as well as in the right precuneus (*r* = −0.295, *p* = 0.009). Additionally, a significant positive correlation was found between ALFF and GMV in the left FFG (*r* = 0.280, *p* = 0.010) and left putamen (*r* = 0.321, *p* = 0.006). However, these significant correlations were not present in the NH group.

**Table 4.** The correlation between ALFF and GMV in covariant areas of the presbycusis group

Covariant areas	*r* value	*p* value	*p* value (FDR)
Medial superior frontal gyrus	−0.320	0.003	0.006*
Precuneus	−0.295	0.007	0.009*
Fusiform gyrus	0.280	0.010	0.010*
Putamen	0.321	0.003	0.006*

FDR corrected *p* < 0.05. The bold and asterisked (*) values are statistically significant. ALFF, amplitude of low-frequency fluctuation; GMV, gray matter volume.

### Functional-structural coupling analysis

**Figure 3.**
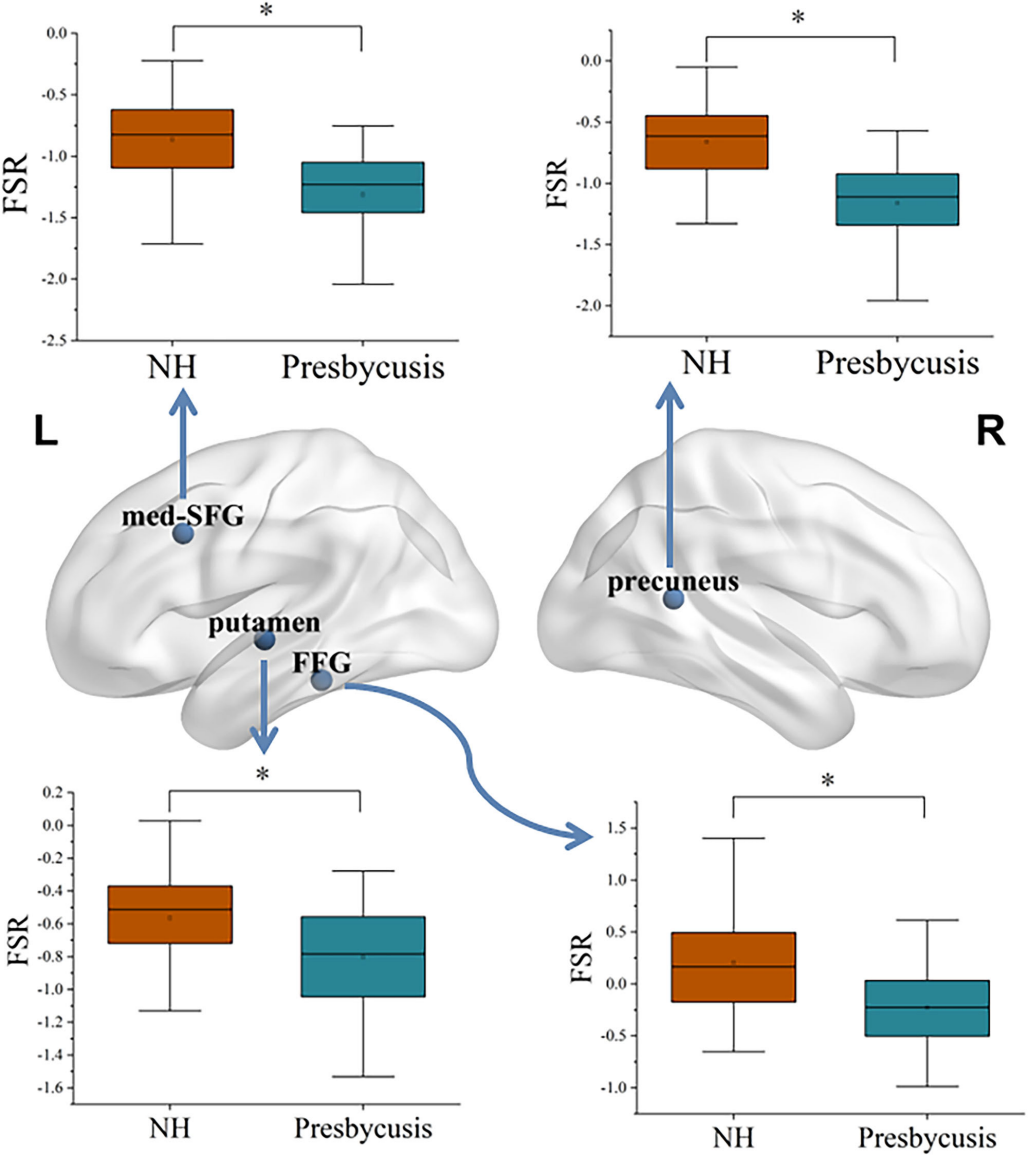
Statistical differences chart of the FSR between presbycusis and NH groups. * represents Bonferroni’s correction, *p* < 0.01. FSR, functional-structural ratio; med-SFG, medial superior frontal gyrus; FFG, fusiform gyrus.

**Figure 4.**
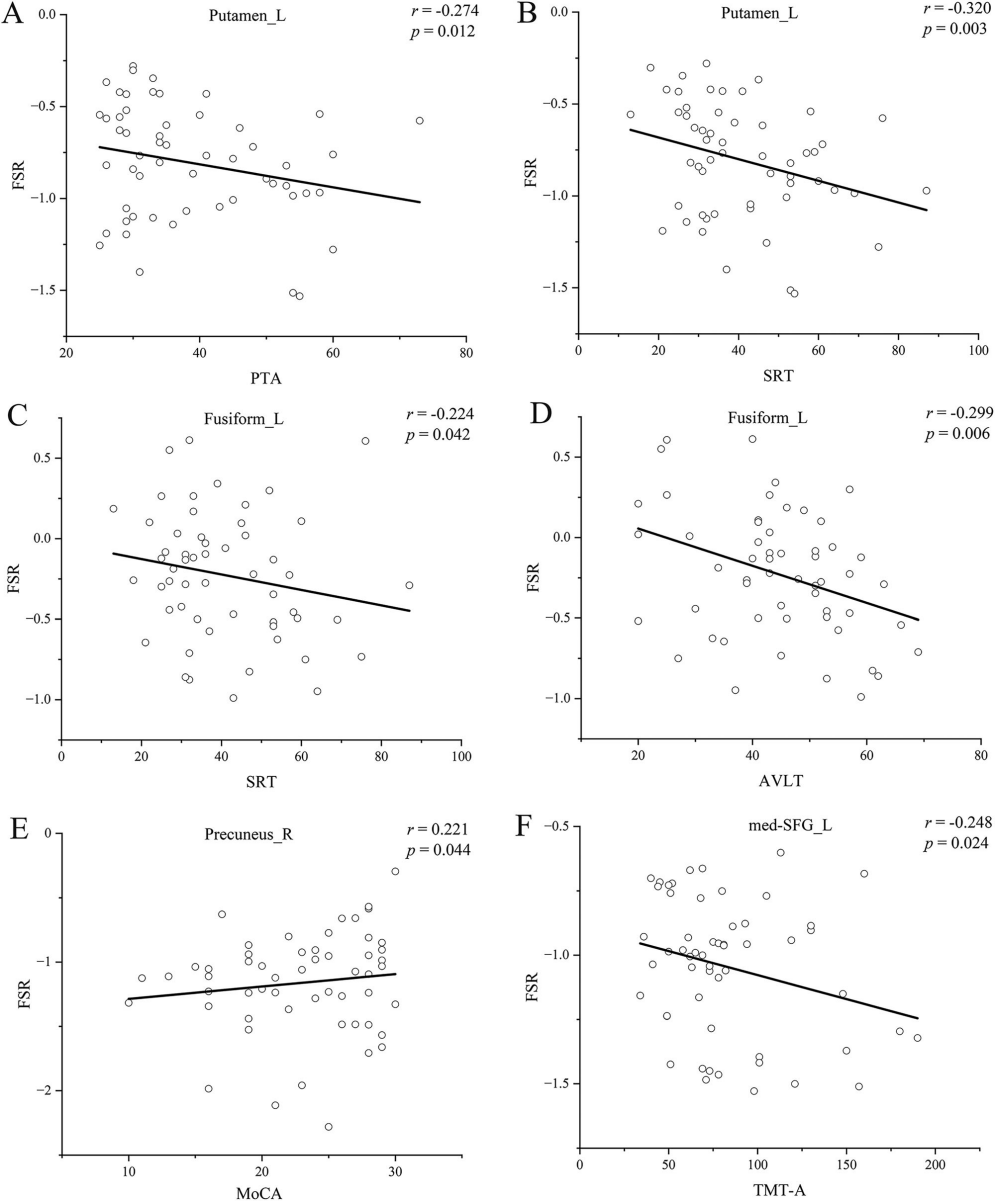
***A–F***, Correlations between the FSR and behavioral score in the presbycusis. FSR, functional-structural ratio; PTA, pure tone average; SRT, speech reception threshold; MoCA, Montreal Cognitive Assessment; AVLT, Auditory Verbal Learning Test.

Figure 3 shows the statistical difference in FSR between the presbycusis and NH groups. Significant intergroup differences in FSR were found in the left FFG, med-SFG, precuneus, and right putamen. Specifically, the left med-SFG, putamen, FFG, and right precuneus all showed reduced FSR in the presbycusis when compared with the NH controls. The correlation between FSR and clinical variables in the presbycusis group is illustrated in Figure 4. In the right putamen, there was a significant negative correlation between FSR and PTA (*r* = −0.274, *p* = 0.012), as well as SRT (*r* = −0.320, *p* = 0.003). Similarly, in the left FFG, there were negative correlations observed with SRT (*r* = −0.224, *p* = 0.042) and AVLT (*r* = −0.299, *p* = 0.006). On the other hand, a positive correlation was found between FSR in the right precuneus and MoCA scores (*r* = 0.221, *p* = 0.044). Lastly, a negative correlation was observed between FSR in the right med-SFG and TMT-A performance (*r* = −0.248, *p* = 0.024).

## Discussion

To the best of our knowledge, this study is the first study to examine regional functional-structural coupling (FSR = ALFF/GMV) in presbycusis by integrating sMRI and fMRI. We observed widespread GMV reductions accompanied by regionally heterogeneous changes in ALFF. Importantly, GMV and ALFF alterations overlapped in the putamen, FFG, precuneus, and med-SFG. In presbycusis, the putamen and FFG exhibited synchronous reductions in both ALFF and GMV. The precuneus and med-SFG showed increased ALFF alongside GMV loss. Lower FSR in the putamen correlated with worse hearing loss and speech recognition, while reduced FSR in the FFG was linked to worse speech recognition. We also observed region-specific FSR reductions were related to clinical and cognitive measures: lower FSR in the FFG was linked to poorer AVLT performance, consistent with a role for functional-structural alterations in verbal episodic memory decline; reduced FSR in med-SFG was associated with poorer performance on the TMT-A, which we interpret as indicative of altered executive function; and lower FSR in the precuneus correlated with reduced total MoCA scores, reflecting poorer general cognitive functioning.

We observed widespread cortical atrophy in presbycusis, together with regional alterations in spontaneous neural activity—some regions showed increases while others showed decreases. Prior studies have reported similar patterns of atrophy in regions such as the HG, STG, precuneus, and SMA (Ren et al., 2018) and mixed ALFF alterations in presbycusis (Chen et al., 2018), which have been interpreted as simultaneous disruption and compensation responses (Tarawneh et al., 2022). In our covariant regions, ALFF and GMV were significantly correlated, suggesting partial interdependence of structural and functional alterations in presbycusis. Consistent with our findings, a previous study has reported correlations between ALFF and GMV within the posterior cingulate cortex in mild cognitive impairment patients (Z-L. Zhao et al., 2015). It is also consistent with a large-scale finding of concurrent hippocampal volume reduction and decreased hippocampus-HG functional connectivity in presbycusis (Fitzhugh and Pa, 2022). Notably, the regional specificity of ALFF and GMV alterations observed in our study may indicate a nonlinear relationship between functional and structural damage. This notion resonates with staged models of disease progression—early reversible fluctuations, compensatory changes, mid-late decompensation, and late structural decline (Lou et al., 2016; Li et al., 2021; Liu et al., 2021). While evidence suggests functional and structural remodeling in sensorineural hearing loss may follow different stages (Uchida et al., 2011), their precise interplay requires further clarification through animal and longitudinal studies. Our findings are consistent with the sensory deprivation hypothesis, wherein partial auditory deprivation precipitates cortical functional decline and subsequent cognitive deficits. However, we cannot rule out that preexisting brain changes or cognitive impairment contribute to the observed ALFF and GMV alterations, as clinical investigations have not addressed the causal link between auditory deprivation in presbycusis and cognitive decline.

Our study revealed that presbycusis exhibits reduced ALFF and GMV atrophy in the left putamen. The putamen is structurally interconnected with the thalamus and is believed to play a crucial role in language processing (Viñas-Guasch and Wu, 2017). In line with our findings, previous study has reported reduced ALFF in the putamen within the slow-4 frequency band in presbycusis (Ren et al., 2021), while another study reported putamen atrophy in patients with sensorineural HL (Qu et al., 2020). Notably, we observed disrupted functional-structural coupling in the left putamen in presbycusis, which was correlated with hearing loss and speech recognition proficiency. The left putamen contributes to speech comprehension (Abutalebi et al., 2013), and its neurons encode auditory information (Z. Zhao et al., 2015). Taken together, these findings suggest putamen reorganization after auditory deprivation may impair auditory encoding and contribute to reduced speech recognition in presbycusis.

Impairments in functional-structural coupling in the FFG were associated with diminished speech recognition proficiency in presbycusis. The FFG, located in the lateral temporal lobe, contributes to recognition processes and provides top-down feedback important for speaker recognition (Rhone et al., 2023). We also found that the altered coupling in the FFG is associated with poorer learning and memory in presbycusis. Although a direct link between FFG impairment and memory ability remains unestablished, previous research has revealed a distinct association between working memory and activation in the FFG (Owens et al., 2018). Given the crucial role of the FFG in audiovisual speech processing (McNorgan and Booth, 2015), altered coupling may reflect changes in audiovisual integration. Audiovisual information can interfere with each other during brain processing, thereby impacting working memory (He et al., 2023). Supporting this, several studies report enhanced information transfer from the FFG to other GM regions and increased audiovisual integration abilities in presbycusis (Schulte et al., 2020; Rosemann et al., 2021; Chavant and Kapoula, 2022). Consistent with this view, a recent study reported a unique association between spontaneous neural activity on the FFG and the duration of HL in sensorineural hearing loss patients (Li et al., 2023). Collectively, these observations suggest that the FFG may participate in an audiovisual compensatory process in presbycusis that is modulated by the chronicity of hearing loss.

We also found that impaired functional-structural coupling in the precuneus was present in presbycusis and was associated with general cognitive impairments. The precuneus is connected to the STG via the middle longitudinal fasciculus and is involved in the integration of auditory information (Tanglay et al., 2022). It is also implicated in various cognitive processes, including self-awareness, episodic memory retrieval, and spatial information processing (Ponticorvo et al., 2021; Dadario and Sughrue, 2023). In addition, presbycusis was linked to disrupted functional-structural coupling in the med-SFG, and weaker coupling in this region correlated with poorer executive performance. The med-SFG is structurally connected to the anterior cingulate cortex and involved in auditory spatial processing (Tao et al., 2015). The present study revealed a significant association between deactivation in the med-SFG and impaired executive function, which holds true in children, adolescents, and adults (Müller et al., 2021). It is noteworthy that both the precuneus and the med-SFG serve as pivotal nodes within the default mode network (DMN). Previous studies have reported atrophy of internal nodes within DMN and reduced functional connectivity in presbycusis (Ren et al., 2018; Xing et al., 2020). Together, these results indicate that partial auditory deprivation in presbycusis is accompanied by DMN reorganization, which may contribute to declines in multiple cognitive domains.

This study found that presbycusis exhibits a functional-structural coupling relationship in the FFG, med-SFG, putamen, and precuneus. The auditory deprivation hypothesis proposes a causal relationship between auditory impairment and changes in brain structure caused by auditory deprivation (Slade et al., 2020). The sensory deprivation hypothesis explains the compensatory reorganization of cortical function and the cognitive regulation of presbycusis. Furthermore, a study has hypothesized that brain functional reorganization may occur earlier than structure (De Micco et al., 2021). This study found a possible association between brain structure and function, despite the lack of clinical evidence to support a causal relationship between the two. In addition, presbycusis exhibits a relationship between functional-structural coupling and cognitive impairments, which provides a new supplement to the hypothesis of sensory deprivation.

There are several limitations to this study that need to be noted. First, because this study adopted a cross-sectional design, the observed associations between structural and functional alterations in presbycusis do not establish causality or temporal order. It remains unclear whether hearing loss leads to neural and cognitive changes or whether preexisting brain alterations contribute to auditory dysfunction. Residual confounding and reverse causation therefore cannot be excluded, and longitudinal or interventional studies will be needed to clarify the direction of these effects. Second, it is also constructive to carry out an intergroup difference study after first establishing the relationship between structure and functionality. Future studies can try to actualize this ideal at the structural and functional network levels. Third, due to the limited sample size, subgrouping participants based on their cognitive abilities was not performed. Therefore, the altered function and structure observed in presbycusis may also be attributed to cognitive impairment. In the future, it is necessary to expand the sample size for subgroup analysis to further clarify which functional reorganizations in presbycusis are caused by hearing impairment. Finally, while FSR highlighted region-specific decoupling in our sample, we emphasize that other coupling metrics (regression, covariance, canonical correlation, joint ICA, etc.) may reveal complementary aspects of structure–function relationships. Ratio measures can be sensitive to measurement noise and scaling differences; therefore, independent-cohort replication and method comparison are essential. We plan to validate FSR findings using (1) regression-based modeling (ALFF ∼ GMV + covariates), (2) multivariate fusion methods (e.g., joint ICA/linked ICA), and (3) replication in larger/independent datasets.

## Conclusion

Our findings suggest that functional and structural reorganization occurs in presbycusis, with these two aspects being mutually dependent. Notably, impaired speech recognition in presbycusis is correlated with reorganization in specific brain regions (putamen, FFG), which may be primarily established through hearing loss. Furthermore, the association between FFG damage and memory ability implies a reshaping of audiovisual integration in presbycusis. Additionally, our study reveals that presbycusis exhibits functional and structural changes in DMN nodes, which are linked to cognitive impairments across multiple domains.

## Synthesis

Reviewing Editor: Luke Remage-Healey, University of Massachusetts Amherst Decisions are customarily a result of the Reviewing Editor and the peer reviewers coming together and discussing their recommendations until a consensus is reached. When revisions are invited, a fact-based synthesis statement explaining their decision and outlining what is needed to prepare a revision will be listed below. The following reviewer(s) agreed to reveal their identity: Lane Krejcova.

SYNTHESIS:

Two expert reviewers evaluated the manuscript and found that it was innovative and clinically relevant, introducing a potentially useful biomarker of structure-function coupling in presbycusis. That said, they raised several important concerns below that need to be addressed in order to proceed with an evaluation.

REVIEWER COMMENTS:

The manuscript investigates the relationship between presbycusis and cognitive decline through combined structural and functional MRI approaches, introducing the functional-structural ratio (FSR) as a novel biomarker. It is well designed and provides impactful insights into the neurobiological mechanisms linking hearing loss and cognitive decline. The FSR captures the coupling between gray matter volume and spontaneous neural activity. Results show that reduced FSR in the fusiform gyrus, putamen, precuneus, and medial superior frontal gyrus correlates with both worse hearing thresholds and poorer cognitive performance.

POINTS TO BE ADDRESSED

1. It would be important to clarify in the discussion that causality cannot be inferred due to the cross-sectional design.

2. Consider expanding the sample size or performing subgroup analysis for future work to disentangle the relative contributions of hearing impairment vs cognitive decline. The presbycusis group includes individuals with normal cognition, MCI, and dementia. Without subgroup analyses, it is difficult to determine whether findings are driven by hearing loss itself or by underlying neurodegeneration. Stratified analyses or at least sensitivity checks would add clarity.

3. Develop a visual abstract that integrates structural and functional findings with cognitive results for clarity.

4. FSR is defined as ALFF/GMV, but the rationale for this ratio as a biologically meaningful measure of coupling requires further justification. Why this metric over alternative coupling approaches (e.g., regression or covariance analyses)? Validation in an independent cohort or at least comparison to other metrics would strengthen confidence.

5. The fMRI scan duration was only ~2 minutes, which is relatively short for reliable ALFF estimation. This limitation should be explicitly acknowledged, and its implications for reproducibility discussed.

6. The use of primarily screening-level measures (MoCA, AVLT, TMT) limits the resolution of domain-specific inferences. The authors should interpret claims about executive function, memory, and general cognition with appropriate caution.

## Author Response

Response to decision:

1. It would be important to clarify in the discussion that causality cannot be inferred due to the cross-sectional design.

Thank you for highlighting the issue of causal inference. We have revised the Limitations paragraph in the Discussion to explicitly state that, because this is a cross-sectional study, our findings do not establish causality or temporal order. We now note that it is unclear whether hearing loss causes neural and cognitive changes or whether preexisting brain alterations contribute to auditory dysfunction, and that residual confounding and reverse causation cannot be excluded. We also state that longitudinal and interventional studies are needed to clarify the directionality of these effects. (See Discussion, Limitations paragraph.) 2. Consider expanding the sample size or performing subgroup analysis for future work to disentangle the relative contributions of hearing impairment vs cognitive decline. The presbycusis group includes individuals with normal cognition, MCI, and dementia. Without subgroup analyses, it is difficult to determine whether findings are driven by hearing loss itself or by underlying neurodegeneration. Stratified analyses or at least sensitivity checks would add clarity.

Thank you for this important comment. We agree that subgroup analyses could help disentangle the relative contributions of hearing impairment and cognitive decline. As noted in the manuscript (Discussion, Limitations), "Lastly, due to the limited sample size, subgrouping participants based on their cognitive abilities was not performed. Therefore, the altered function and structure observed in presbycusis may also be attributed to cognitive impairment. In the future, it is necessary to expand the sample size for subgroup analysis to further clarify which functional reorganizations in presbycusis are caused by hearing impairment.". To clarify for the Reviewer, the presbycusis group in the present sample comprises dementia : MCI : cognitively normal = 10 : 26 : 19. These subgroup sizes are small and accordingly underpowered for stratified statistical comparisons; performing and reporting such subgroup tests at this stage would risk producing unreliable or misleading results. For this reason, we did not conduct formal subgroup analyses in the current revision. We have therefore interpreted our results at the group level and explicitly acknowledged this limitation in the manuscript. We plan to recruit larger samples and collect longitudinal and interventional data in future studies to enable adequately powered subgroup and interaction analyses that can more robustly disentangle hearing-related vs. neurodegeneration-related effects.

3. Develop a visual abstract that integrates structural and functional findings with cognitive results for clarity.

Thank you for the suggestion. We have developed a visual abstract that integrates clinical measures (hearing and cognition), imaging findings (ALFF and GMV), and the FSR results with arrows illustrating the principal associations.

4. FSR is defined as ALFF/GMV, but the rationale for this ratio as a biologically meaningful measure of coupling requires further justification. Why this metric over alternative coupling approaches (e.g., regression or covariance analyses)? Validation in an independent cohort or at least comparison to other metrics would strengthen confidence.

Thank you for raising this important methodological point. In response, we have strengthened the manuscript in two ways. First, we added a paragraph to the Introduction that motivates the use of ratio-based coupling indices (e.g., ALFF/GMV), cites recent relevant work, and explains the conceptual interpretation of such measures as "neural activity demand per unit gray-matter volume." Second, we explicitly acknowledged the limitations of ratio metrics in the Discussion and committed to future method comparisons and validation: we will compare FSR with regression/covariance and multivariate fusion approaches (e.g., joint/linked ICA or canonical correlation) and attempt independent-cohort replication. These additions should clarify the rationale, implementation, and limitations of the FSR metric. (See Introduction and Discussion.) 5. The fMRI scan duration was only ~2 minutes, which is relatively short for reliable ALFF estimation. This limitation should be explicitly acknowledged, and its implications for reproducibility discussed.

Thank you for the comment. We discovered that the "~2 minutes" statement in the original manuscript was a typographical error. The resting-state fMRI acquisition was in fact 8 minutes (TR = 2 s; 240 volumes; total duration = 480 s). We have corrected this in the Methods.

6. The use of primarily screening-level measures (MoCA, AVLT, TMT) limits the resolution of domain-specific inferences. The authors should interpret claims about executive function, memory, and general cognition with appropriate caution.

We agree with the Reviewer. We have revised the Discussion to first report FSR associations at the measurement level and then cautiously infer links to broader cognitive domains. This change clarifies that our claims are based on test-score associations and avoids implying direct, mechanistic relationships between imaging markers and specific cognitive subcomponents.
